# Neither Invasive nor Unprotected: The Legal Status of Muscovy Ducks in the United States

**DOI:** 10.3390/ani16213472

**Published:** 2026-11-03

**Authors:** Anne Steinemann

**Affiliations:** College of Science and Engineering, James Cook University, Townsville, QLD 4811, Australia; drsteinemann@gmail.com

**Keywords:** Muscovy Duck, *Cairina moschata*, non-native species, invasive species, introduced species, anti-cruelty, animal welfare, wildlife classification, wildlife law

## Abstract

Muscovy Ducks (*Cairina moschata*), one of the world’s oldest domesticated waterfowl, inhabit nearly all continents. Considered non-native across most of the United States, Muscovy Ducks are often described as “unprotected” or “invasive,” terms that may be used to justify harmful actions. The question is whether these descriptions accurately reflect their legal protection and wildlife classification. This study addresses that question through the first nationwide analysis of anti-cruelty statutes and invasive-species classifications at the federal and state levels. Results show that every state prohibits intentional cruelty toward Muscovy Ducks, subject to exemptions. While considered “unprotected” for conservation purposes, they remain protected under anti-cruelty statutes. No federal agency formally classifies Muscovy Ducks as “invasive.” Although two of the 50 states list Muscovy Ducks as invasive, each listing lacks science-based substantiation of harm in the state. This study corrects claims that Muscovy Ducks are legally unprotected or invasive. Misuse and misinterpretation of terms can obscure the reality that intentional cruelty against Muscovy Ducks is unlawful nationwide.

## Simple Summary

Muscovy Ducks (*Cairina moschata*) live alongside people in communities around the world, as they have for centuries. Across most of the United States, they are considered non-native and often called “unprotected” or “invasive.” These labels can influence how people perceive and treat the ducks. This study examines whether those labels accurately describe the legal and wildlife status of Muscovy Ducks. Through a nationwide analysis, this research investigates animal-cruelty laws and invasive-species classifications at both federal and state levels. Results reveal a disconnect between common labels and legal reality. Every state has criminal laws that protect Muscovy Ducks from intentional cruelty, subject to exemptions. No federal agency formally classifies Muscovy Ducks as invasive. Among the 50 states, two list Muscovy Ducks as invasive, but each listing lacks science-based substantiation of harm in the state. “Non-native” itself does not fulfill the harm-based criteria of invasive status. These findings correct common descriptions of the legal protection and invasive status of Muscovy Ducks. Being non-native does not mean invasive, and being unprotected for conservation purposes does not mean unprotected from cruelty. When these terms are used loosely, they can make harmful treatment seem acceptable or justified. Rectifying this rhetoric is important to help prevent misunderstanding and mistreatment.

## 1. Introduction

Long domesticated, relatively calm, usually silent, and accustomed to humans, Muscovy Ducks (*Cairina moschata*) have adapted to life alongside people [1,2,3]. The native range of the Muscovy Duck extends from northern Argentina through South America, Central America, and Mexico to southern Texas [3]. Outside this range, non-native (introduced) Muscovy Ducks are found as domesticated and feral populations around the world [3,4].

In the United States, especially in the state of Florida, Muscovy Ducks are a familiar presence in human-settled environments with water bodies, such as residential communities, recreational facilities, golf courses, and municipal parks with ponds, canals, and wetlands. Feral and free-ranging populations can become dependent on humans for survival, especially through food handouts [5]. This combination of familiarity and dependence places Muscovy Ducks in frequent and unusually direct contact with humans.

Despite their human-adapted behaviour, Muscovy Ducks have been subjected to reported acts of intentional harm and lethal cruelty, including beating, deliberate vehicle strikes, drowning, poisoning, and shooting with arrows (e.g., [6,7,8]). While the purpose of this study is not to investigate these reports, they nonetheless motivate inquiry into the legal status of Muscovy Ducks.

Muscovy Ducks are frequently described in public and institutional discourse as “unprotected” or “invasive.” These terms have distinct legal and policy meanings and can influence public perception and treatment of animals. Yet it remains unclear whether these commonly used descriptions accurately represent legal protection and invasive-species status of Muscovy Ducks in the United States.

This study investigates the central research question: How are Muscovy Ducks treated under animal law and wildlife classifications? More specifically, are Muscovy Ducks, as a non-native species, in fact legally unprotected and invasive?

Terms such as “unprotected” or “invasive” may be conflated with “non-native,” although they represent distinct concepts. Non-native status concerns the geographic origin of a species or population. A non-native species may be “unprotected” under a conservation framework, yet protected under anti-cruelty legislation. A non-native species is not “invasive” without a science-based determination of actual or likely harm for the ecosystem under consideration [9,10]. Additionally, non-native status is not an animal-welfare status. Non-nativity does not lessen an individual animal’s experience of distress, fear, pain, or suffering.

The literature converges on three points relevant to the present study. First, labels carry meaning and can influence how animals are perceived and treated. Terms such as “invasive,” “non-native,” and “pest” have been associated with negative framing and greater acceptance of lethal control or eradication [11,12]. Militaristic metaphors can portray targeted species as enemies to be defeated or eliminated [13], and present cruelty as patriotism [14]. Government use of these labels can make the killing of animals appear justified, acceptable, and even responsible [15]. Muscovy Duck news narratives in Florida and Texas portrayed the bird as either “invasive” and in need of removal, or as “family” and in need of protection [6].

Second, labels can be used without adequate substantiation or precision. Variable and inaccurate use of invasive-species terminology can create confusion [16], and be manipulated to suit an agenda [11]. The juxtaposition of “non-native” and “invasive” can suggest that species are harmful because they are non-native [17]. In Florida news coverage, the perceived status of Muscovy Ducks as “invasive” or “introduced” has been associated with policies that promote lethal population control [7,8]. Management decisions should be based on scientific evidence of significant harm rather than on negative labels or geographic origin [18,19].

Third, labels used for species or populations can obscure the welfare interests of individual animals. Animals assigned negative labels can receive less welfare consideration, even though welfare consideration should not depend on how a species is classified [18]. Classification as a “pest” can affect the application of anti-cruelty law despite the suffering inflicted [20]. Cruelty can also be normalised in the name of conservation [21]. For Muscovy Ducks, their perceived status as an “invasive nuisance” can make them vulnerable to acts of cruelty [6,7]. The suffering of individual animals remains morally relevant regardless of species membership [22,23,24,25].

This study provides the first nationwide analysis of legal protections and wildlife classifications for Muscovy Ducks. It examines anti-cruelty statutes and invasive-species classifications at the federal level and for each of the 50 states. Muscovy Ducks provide an instructive case to critically examine whether prominent narratives describing them as “unprotected” and “invasive” are supported by the underlying legal and policy record.

## 2. Materials and Methods

A structured legal and policy analysis was conducted at both federal and state levels to assess (1) whether Muscovy Ducks are protected under anti-cruelty statutes, and (2) whether Muscovy Ducks are formally classified as invasive. The research was conducted from 2025 to 2026, with the most recent verification of statutes in August 2026.

The federal analysis (Section 3.1.1) first examined the statutes and regulations governing Muscovy Ducks, specifically the Migratory Bird Treaty Act and the Muscovy Duck Control Order. This examination provided the framework for the subsequent analyses of anti-cruelty protections and invasive-species status.

The federal anti-cruelty analysis (Section 3.1.2) examined federal statutes relevant to animal protection and humane treatment to identify protections applicable to Muscovy Ducks. The examination considered the scope of each statute, relevant definitions, prohibited conduct, and applicable exemptions.

The federal invasive-species analysis (Section 3.1.3) examined the applicable federal definition of invasive species, federal agency lists and databases, and other official agency materials. The search covered federal agencies with jurisdiction over Muscovy Ducks as well as agencies that provide national information on invasive species.

The invasive-species status of the Muscovy Duck was investigated using a two-step analytic approach: First, the study determined whether the Muscovy Duck was formally listed as an invasive species by the relevant agency. This first step distinguished formal administrative designations or listings from colloquial or descriptive usages of the term invasive. Second, if such a listing was identified, the study evaluated whether the listing was substantiated with a formal science-based assessment that the species causes or is likely to cause harm in the ecosystem under consideration. This second step evaluated the science-based substantiation for the listing. This analytic approach reflects federal guidance that invasive-species determinations should be based on a context-specific, science-based assessment of actual or likely harm [10,26]. To verify or clarify the designation or substantiation, in cases where the public record was not definitive, the designated officials at the relevant government agencies were contacted directly via formal email inquiry.

The anti-cruelty analysis, for each of the 50 states (Section 3.2.1), examined the principal animal-cruelty statute in its entirety, including statutory provisions, cross-references, definitions, scope of protection, prohibited conduct, offense classifications, exclusions, and exemptions. Statutes were retrieved and verified utilizing public state legislature databases. Where the principal animal-cruelty statute did not fully address the protection of Muscovy Ducks, the analysis extended to relevant provisions in other statutes. The analysis focused on whether Muscovy Ducks fall within the scope of anti-cruelty protections, through one or more statutory provisions, considering relevant definitions, exclusions, and exemptions. It did not seek to assess the enforcement and judicial interpretation of these statutes, applicability of state wildlife-management laws, or protection against all possible acts or activities. The anti-cruelty statutes examined are summarized in Appendix A, with detailed state-by-state determinations provided in Supplementary File S1 and discussed in Section 3.2.1.

The invasive-species analysis, for each of the 50 states (Section 3.2.2), first identified the primary state agency responsible for invasive-species classification. Unlike state anti-cruelty laws, invasive-species classifications are not necessarily contained in a single consolidated statute or regulation. States differ considerably in how they classify invasive species and where they publish their species lists, making them difficult to locate. Accordingly, the search proceeded from the relevant state agency, examining statutes, regulations, official agency databases, administrative codes, species registries, and other classification materials. The analysis followed the two-step analytic approach described for the federal analysis. To verify or clarify the designation or substantiation, in cases where the public record was not definitive, the relevant agencies were contacted directly via formal email inquiry. The invasive-species agencies examined are summarized in Appendix A, with detailed state-by-state determinations provided in Supplementary File S2 and discussed in Section 3.2.2. 

## 3. Results

### 3.1. National Framework

#### 3.1.1. Federal Statutes and Regulations

Muscovy Ducks are listed as protected migratory birds under the Migratory Bird Treaty Act (MBTA) (16 U.S.C. §§ 703–712 [27]; 50 C.F.R. § 10.13 [28]). The MBTA makes it unlawful to “take, capture, kill, attempt to take, capture, or kill” any migratory bird covered by the Act, without federal authorization such as a permit or regulation (16 U.S.C. § 703(a)). Under the MBTA, “take” means “to pursue, hunt, shoot, wound, kill, trap, capture, or collect, or attempt to pursue, hunt, shoot, wound, kill, trap, capture, or collect” (50 C.F.R. § 10.12).

Protection under the MBTA is granted to birds that are native, defined as “occurring in the United States or its territories as the result of natural biological or ecological processes” but not “solely as a result of intentional or unintentional human-assisted introduction” (16 U.S.C. § 703(b)(2)(A)–(B)).

Muscovy Ducks occur both as a native population, in southern Texas (Hidalgo, Starr, and Zapata Counties) (p. 9296, [5,29]), and as an introduced population elsewhere in the United States. Thus, Muscovy Ducks do not occur “solely” as a result of introduction. By virtue of their native population in southern Texas, all Muscovy Ducks in the U.S. receive the protection of the MBTA (75 Fed. Reg. 9296 (1 March 2010) [5]).

On the same day that the Muscovy Duck received nationwide protection under the MBTA, the U.S. Fish and Wildlife Service implemented the Muscovy Duck Control Order, which states that anywhere in the contiguous United States except in the native range, “landowners and Federal, State, Tribal, and local wildlife management agencies, and their tenants, employees, or agents may, without a Federal permit, remove or destroy Muscovy ducks (*Cairina moschata*) (including hybrids of muscovy ducks), or their nests, or eggs at any time when found” (50 C.F.R. § 21.54(a) (2010) [30]).

Non-native Muscovy Ducks are not inherently “unprotected” under the MBTA. They remain MBTA-listed birds, but the Muscovy Duck Control Order removes the ordinary federal permit requirement for “take” outside their native range. In this sense, Muscovy Ducks may be regarded as “unprotected” for conservation purposes, meaning they can be killed without a federal permit, but not “unprotected” from penal law, such as state anti-cruelty protections.

The Muscovy Duck Control Order requires compliance with “any State, territorial, or Tribal laws or regulations governing the removal or destruction of Muscovy ducks or their nests or eggs” (50 C.F.R. § 21.174(d)(1) [31]). The MBTA does not preclude states from “making or enforcing laws or regulations which shall give further protection to migratory birds, their nests, and eggs” (16 U.S.C. § 708). Thus, the Muscovy Duck Control Order does not displace otherwise applicable state anti-cruelty laws.

The Muscovy Duck Control Order is unusually permissive and expansive: it does not require any justification or evidence of harm to kill the ducks, it does not prescribe or restrict methods of killing (other than nontoxic shot or bullets if a firearm is used), it does not provide for non-lethal removal options, it does not require the use of non-lethal methods before lethal control, it does not require routine monitoring or reporting, and it allows ducks to be killed at any time across more than 99% of the contiguous United States [32,33,34] (50 C.F.R. Part 21, Subpart D).

Although not the focus of this paper, the Muscovy Duck Control Order provides critical context for how Muscovy Ducks have been framed, particularly regarding their legal protection and invasive characterization.

#### 3.1.2. Federal Anti-Cruelty Statutes

Federal law provides specific protections rather than a general anti-cruelty framework. Federal protections relevant to Muscovy Ducks include the following. The Preventing Animal Cruelty and Torture (PACT) Act prohibits purposeful “animal crushing in or affecting interstate or foreign commerce or within the special maritime and territorial jurisdiction of the United States” (18 U.S.C. § 48(a)(1) [35]). The Animal Welfare Act (AWA) requires humane treatment of animals in “research, teaching, testing, exhibition, transport, and by dealers” [36] (7 U.S.C. §§ 2132 [37], 2143(a) [38]). The Migratory Bird Treaty Act (MBTA) makes it unlawful to “take, capture, kill, attempt to take, capture, or kill” any migratory bird covered by the Act, but does not govern their treatment (16 U.S.C. § 703(a)). These federal statutes provide protections within their respective statutory scopes and do not establish a general prohibition against cruelty to Muscovy Ducks in all circumstances. Thus, the primary legal frameworks governing the treatment of Muscovy Ducks reside within state animal-cruelty statutes, examined in Section 3.2.1.

#### 3.1.3. Federal Invasive-Species Classification

The federal framework provides a specific and unifying definition of invasive species. Under Executive Order 13,112, an “invasive species” is defined as “an alien species whose introduction does or is likely to cause economic or environmental harm or harm to human health,” where an alien species is “not native to that ecosystem” (64 Fed. Reg. 6183 (1999) [9]). In addition, “for policy purposes, to be considered invasive, the negative impacts caused by a non-native species will be deemed to outweigh the beneficial effects it provides” [10]. Under Executive Order 13,751, which subsequently amended Executive Order 13,112, an “invasive species” is defined as “with regard to a particular ecosystem, a non-native organism whose introduction causes or is likely to cause economic or environmental harm, or harm to human, animal, or plant health” (81 Fed. Reg. 88,609 (2016) [39]). For the key differences, the amendment uses “non-native organism” rather than “alien species,” ties invasive status to a particular ecosystem, and expands health-related harm to include animal and plant health. It retains actual or likely harm as a necessary criterion for invasive status.

At the federal level, Muscovy Ducks are not formally classified as invasive. The species is absent from invasive species lists and databases maintained by the National Invasive Species Information Center [40], the U.S. Fish and Wildlife Service [41], and the U.S. Geological Survey [32,42].

The National Invasive Species Information Center confirmed that “the Muscovy Duck is not considered invasive at a federal level” [43]. The USFWS National Invasive Species Program Coordinator also confirmed, “Muscovy Ducks are not formally classified as an invasive species by the U.S. Fish and Wildlife Service” [44]. The USFWS Acting Chief of the Division of Migratory Bird Management similarly confirmed, “There is no formal designation of Muscovy as invasive” [45]. The U.S. Geological Survey confirmed, “The Muscovy Duck is not shown on the ‘Invasive Species We Study’” [32,46].

Taken together, these federal sources clearly establish that Muscovy Ducks are not formally classified as invasive at the federal level. The analysis now turns to the state level, where both anti-cruelty protections and invasive-species classifications vary by jurisdiction.

### 3.2. State Frameworks

#### 3.2.1. State Anti-Cruelty Statutes

All U.S. states prohibit intentional cruelty toward animals through broadly applicable criminal statutes (Supplementary File S1).

In each of the 50 states, statutory language applies to “any animal,” “an animal,” or similar inclusive terms. To qualify these broad terms, some states (12) provide definitions of animal, such as all living creatures except humans, vertebrates, or specific categories (e.g., mammals, birds, reptiles, amphibians, and fish).

In addition, some states contain wildlife-specific provisions or exclusions. For example, Iowa excludes livestock, including poultry, from its general animal-cruelty provisions (§ 717B.1), but addresses livestock separately (Iowa Code §§ 717.1, 717.1A, 717.2). South Carolina excludes fowl from one anti-cruelty provision (§ 47-1-40), while other provisions protect animals more broadly (§§ 47-1-10, 47-1-50, 47-1-70). Texas has separate anti-cruelty provisions for livestock, including agricultural fowl (§ 42.09), and for nonlivestock animals, including domesticated animals but excluding uncaptured wild animals (§ 42.092). These distinctions were considered in determining whether Muscovy Ducks fall within the scope of state anti-cruelty protections.

None of the statutes exclude species based on classifications such as “non-native,” “invasive,” “introduced,” or “feral.” The anti-cruelty laws establish protection of animals independent of their wildlife classification.

Prohibited conduct across state statutes includes terms such as torture, injure, beat, poison, torment, neglect, and cause pain or suffering. Common qualifiers for these terms include intentional, unnecessary, unjustifiable, malicious, and cruel. Although the term “kill” appears in 36 state statutes, it is generally subject to qualifiers such as “unnecessarily.” While some statutes prohibit killing an animal “without consent of owner,” this qualifier does not necessarily make ownership a prerequisite for protection against cruel killing. Furthermore, other prohibited acts of cruelty identified in these statutes are not conditioned on ownership, and feral Muscovy Ducks remain protected.

Key terms relevant to Muscovy Ducks are provided in Supplementary File S1. Terms specific to other animals, such as overloading and overworking, are not included.

Nearly all states include exemptions for activities such as veterinary care, animal husbandry, hunting, fishing, pest control, livestock practices, rodeo, and scientific research. These exemptions can limit the protection afforded by anti-cruelty laws. An act that constitutes cruelty outside an exempt activity may not be prohibited when it occurs within that activity. Accordingly, the protection of Muscovy Ducks within the scope of an anti-cruelty statute does not establish protection against every act of cruelty. However, activities that involve killing must still comply with applicable state and federal laws.

All states contained felony provisions for severe acts of animal cruelty. In most of the states, these provisions were explicitly identified in anti-cruelty statutes, while in other states, they appeared through criminal classifications, aggravated cruelty statutes, or separate penalty sections. Thus, severe acts of cruelty are treated as serious criminal offenses.

The analysis for each of the 50 states found that their anti-cruelty statutes protect Muscovy Ducks through one or more applicable provisions. Even though exemptions may limit the applicability of anti-cruelty protections to certain activities, none of the states exclude Muscovy Ducks from all protection.

#### 3.2.2. State Invasive Species Classification

Among the 50 states, 48 states do not provide an administrative designation or formal listing of Muscovy Ducks as invasive. Two states do, however, provide a formal listing: New York and Louisiana. New York lists the Muscovy Duck as a “regulated invasive species” under NYS Regulation 6 NYCRR Part 575. Louisiana lists the feral Muscovy Duck as a “Tier 3 invasive species” in its 2025 State Wildlife Action Plan. However, in the study’s evidentiary evaluation of the administrative designation, each of these listings lacked formal science-based substantiation that Muscovy Ducks cause harm in the state.

New York defines “invasive species” as “a species that is nonnative to the ecosystem under consideration, and whose introduction causes or is likely to cause economic or environmental harm or harm to human health. For the purposes of this Part, the harm must significantly outweigh any benefits” (6 CRR-NY 575.2(s) [47]). A “regulated invasive species” means “a species that has the potential to cause significant harm to New York’s economy, ecological well being and/or human health….” (6 CRR-NY 575.2(ah) [47]).

However, the ecological and socio-economic assessments to support the listing of the Muscovy Duck as “invasive” did not provide scientific evidence that established harms in New York [48,49]. The sources cited for evidence were general references or relevant to Florida. Also, the ecological assessment stated that Muscovy Ducks were “Not known to be established” in natural areas of New York, “Currently, feral population in the NE are extremely limited,” and “Populations in the Northeast are extremely limited.” Yet it assigned scores for “Significant alteration of ecosystem processes” and other harms without evidence documenting those effects in New York. When asked how the Muscovy Duck fulfilled the statutory harm-based requirements for an invasive classification, the New York Department of Environmental Conservation explained that the listing of the Muscovy Duck under §575.5 is “not bound solely” to the statutory invasive-species definition [50].

Louisiana defines “invasive species” as “non-native species that have become established in an area, causing negative impacts to native species and their environment” (p. 182, [51]). The Louisiana Department of Wildlife and Fisheries (LDWF) listed the Muscovy Duck “in Tier 3, in part, because the full impacts of the species are not well resolved in Louisiana and because where the species does occur, impacts appear to be present but limited (at this time).” LDWF confirmed that it “did not conduct a formal assessment of harm to support the classification of the Muscovy Duck as invasive and its placement in Tier 3” [52].

Thus, New York has a regulated invasive species listing and Louisiana has a departmental Tier 3 invasive species listing. But neither state identified a formal science-based substantiation that Muscovy Ducks cause harm in the state. These findings distinguish the administrative designation of a species as invasive from the science-based substantiation to support that designation.

## 4. Discussion

This nationwide analysis establishes two main findings. First, Muscovy Ducks are protected from intentional cruelty under the laws of all fifty states. Second, no federal agency formally classifies them as invasive. At the state level, New York and Louisiana list Muscovy Ducks as invasive, but without formal science-based substantiation of harm.

Muscovy Ducks are considered non-native across most of the United States, but non-native status does not itself establish invasive status. The federal rule allowing Muscovy Ducks to be lethally removed outside their native range does not eliminate the protection afforded by state anti-cruelty statutes. None of the fifty state statutes excludes animals from protection because they are non-native, or any other related wildlife classification, such as introduced, feral, or invasive.

### 4.1. Incorrect Labelling

The terms “unprotected” and “invasive” have been used in ways that do not accurately convey the status of Muscovy Ducks.

As a primary example, the “invasive” label is embedded in the federal rulemaking for the Muscovy Duck Control Order. The USFWS Environmental Action Statement stated, “The muscovy duck is an invasive species” [53]. The 2008 Proposed Rule stated that the rule was “intended primarily to reduce the spread of an invasive species” (73 Fed. Reg. 49,628 (22 August 2008) [54]). The 2010 Final Rule stated that “Control of this species in areas in which it is invasive is the intent of this rulemaking” (75 Fed. Reg. 9318 (1 March 2010) [55]) and that “the muscovy duck is an introduced, invasive species” (75 Fed. Reg. 9319 (1 March 2010) [55]).

However, the USFWS does not formally classify the Muscovy Duck as invasive, as noted in Section 3.1.3. When asked specifically about the use of “invasive” in the Proposed and Final rules for the Muscovy Duck Control Order, the USFWS Acting Chief of the Division of Migratory Bird Management explained that the term in the administrative record was “used as a descriptor vs. the formal definition” [32,45]. Yet the term “invasive” formed part of the stated rationale for the Muscovy Duck Control Order.

Furthermore, that descriptive use of “invasive” in the federal administrative record has had consequences. For example, the National Audubon Society published an article stating that “the U.S. Fish & Wildlife Service officially classifies the Muscovy Duck as an invasive species.” It subsequently issued a “Correction: A previous version of this story incorrectly stated that Muscovy Ducks are officially considered invasive by the U.S. Fish & Wildlife Service. The agency classifies the bird as a non-native introduced species” [56].

In addition, a study of Muscovy Duck behaviour in Florida states that “the Muscovy duck is legally recognized as an invasive species” (p. 1, [57]) and cites the USFWS and the text of the Muscovy Duck Control Order [55]. However, as documented in Section 3.1.3 and above, the USFWS does not formally classify the species as invasive, and used the term “invasive” in the Control Order as a descriptor rather than a formal definition.

Thus, the descriptive use of “invasive” in the Muscovy Duck Control Order has been misinterpreted as a formal designation, and perpetuated in public materials.

The same incongruity appears in Florida. The Florida Fish and Wildlife Conservation Commission (FWC) has stated that “The FWC does not formally classify Muscovy ducks as ‘invasive’” [58]. FWC has also stated that Muscovy Ducks are “protected by anti-cruelty laws in Florida (Chapter 828.12 Florida Statutes)” [58,59]. Thus, FWC does not formally classify Muscovy Ducks as invasive and recognizes their protection under state anti-cruelty law.

However, other government sources in Florida convey a different message. For example, “Muscovy ducks are an invasive species” [60]; “Muscovy ducks are considered an invasive species” [61]; Muscovy Ducks “are considered an invasive species in Florida” [62]; and Muscovy Ducks are “not protected” and “labeled invasive” [63]. University of Florida IFAS Extension likewise referred to the Muscovy Duck as “this invasive species” [64]. These labels can misrepresent both invasive status and legal protection.

### 4.2. Additional Considerations

The legal and policy analysis also raises several additional considerations. One is the distinction among legal status, animal welfare, and moral and ethical concerns surrounding harmful treatment and lethal control. Legal questions ask whether conduct is authorised or prohibited by law. Animal-welfare questions ask whether individual animals experience pain, fear, distress, or suffering and whether the methods are considered humane. Moral and ethical questions ask whether harmful treatment or killing is justified, even if legally permitted or considered humane. Legality does not determine whether conduct is humane or morally justified.

Lethal control can be lawful while lethal cruelty can be unlawful. A Muscovy Duck may be unprotected from lawful killing yet protected from cruel killing. However, statutory exemptions or qualifiers may exclude certain acts of killing from otherwise applicable anti-cruelty protections. Moreover, a killing may be lawful but inhumane, and even a lawful killing called humane may remain morally objectionable. That is, killing may constitute cruelty in a broader ethical sense.

The Muscovy Duck Control Order does not prescribe or restrict killing methods, apart from requiring nontoxic shot or bullets when firearms are used (50 C.F.R. § 21.174 [31]). Consequently, methods can vary in the type, extent, and duration of animal-welfare harms [18]. In addition to welfare concerns, lethal control raises ethical questions such as whether killing is necessary or justified, and whether individual animals receive adequate moral consideration. The present study did not evaluate particular killing methods, their animal-welfare consequences, or the moral and ethical considerations associated with lethal control, but these remain important topics for further inquiry.

Population-level classifications may shape the treatment of individual animals. Wildlife classifications concern species and populations, whereas animal welfare and moral consideration concern individual animals. From the perspective of a Muscovy Duck, being described as native, non-native, invasive, or unprotected does not change its experience of pain, fear, and suffering. Nor does it diminish its moral claim to humane treatment. Even if Muscovy Ducks were formally classified as invasive, and even if anti-cruelty protections were not applicable, they would remain deserving of welfare and moral consideration.

### 4.3. Future Research

Results from this study point to topic areas and questions for future research:

Enforcement Gaps: Why may anti-cruelty protections for Muscovy Ducks fail to translate into effective enforcement or deterrence?

Influence of Labels: To what extent would accurate public understanding of the legal protection and invasive-species status of Muscovy Ducks affect perceptions and treatment of the birds?

Animal Welfare: How do wildlife classifications for populations affect humane treatment for individual animals, especially for non-native species such as Muscovy Ducks?

Lethal Control and Sub-lethal Cruelty: Does legal permission to kill Muscovy Ducks create a perception that lesser forms of harm or cruelty are also permissible?

Moral Consideration: How might greater moral consideration of individual Muscovy Ducks challenge the justifications for their lethal control?

Accurate Communication: How can government agencies and other institutions communicate species status accurately and in ways that do not permit or encourage harmful actions?

Anti-Cruelty Protections: How do exemptions limit the applicability of anti-cruelty protections to Muscovy Ducks killed under the federal Muscovy Duck Control Order?

Invasive-Species Classifications: To what extent are invasive-species classifications based on scientific evidence of actual or likely harm for the ecosystem under consideration, rather than assumptions associated with non-native status?

Interspecies Comparisons: How are Muscovy Ducks treated in comparison to other similar species?

International Comparisons: How are Muscovy Ducks classified, protected, and treated in other countries?

## 5. Conclusions

This study sets the record straight on Muscovy Ducks. Muscovy Ducks are protected from intentional cruelty nationwide. Muscovy Ducks are not formally classified as invasive by any federal agency, nor in 48 of the 50 states. While two states list them as invasive, the listings lack science-based substantiation of harm in the state. The label of “invasive” does not substitute for the harm determination that gives the label its substantive meaning.

Being unprotected for conservation purposes does not mean unprotected from cruelty, and being non-native does not mean invasive. Terms such as “invasive,” “non-native,” and “unprotected” can influence how animals are treated, and therefore need to be used with care, especially by public authorities. Findings from this study underscore the importance of correcting terminology that misrepresents the legal protection and invasive-species status of Muscovy Ducks, and that may contribute to mistreatment.

## Data Availability

The data underlying this study consist of publicly available materials, together with correspondence from government officials. Publicly available materials may be accessed through the cited sources; correspondence is available from the author upon request.

## Supplementary Materials

The following supporting information can be downloaded at: https://www.mdpi.com/article/10.3390/ani16213472/s1, File S1: State Anti-Cruelty Statutes for Muscovy Ducks in the United States; File S2: State Invasive-Species Classification and Assessment Status for Muscovy Ducks in the United States.

## Appendix A. Anti-Cruelty Statutes and Invasive-Species Agencies Examined for Muscovy Ducks in the United States

Jurisdiction	Anti-Cruelty Statute(s)/Provisions	Invasive-Species Agency
Federal	18 U.S.C. § 48; 7 U.S.C. §§ 2132, 2143; 16 U.S.C. §§ 703–712	U.S. Fish and Wildlife Service; National Invasive Species Information Center (USDA); U.S. Geological Survey
Alabama	Ala. Code §§ 13A-11-14, 13A-11-14.1	Alabama Dept. of Conservation and Natural Resources
Alaska	AS § 11.61.140	Alaska Department of Fish and Game
Arizona	A.R.S. § 13-2910	Arizona Game and Fish Department
Arkansas	Ark. Code § 5-62-103	Arkansas Game and Fish Commission
California	Cal. Penal Code § 597	California Department of Fish and Wildlife
Colorado	CRS § 18-9-202	Colorado Parks and Wildlife
Connecticut	§ 53-247	Connecticut Dept. of Energy and Environmental Protection
Delaware	§ 1325	Delaware Division of Fish and Wildlife
Florida	§ 828.12	Florida Fish and Wildlife Conservation Commission
Georgia	§ 16-12-4	Georgia Wildlife Resources Division
Hawaii	§ 711-1109	Hawaii Department of Land and Natural Resources
Idaho	§ 25-3504	Idaho Fish and Game
Illinois	510 ILCS 70/3.01, 3.03	Illinois Department of Natural Resources
Indiana	IC § 35-46-3-12	Indiana Division of Fish and Wildlife
Iowa	§§ 717.1A, 717.2, 717B.1–717B.3A	Iowa Department of Natural Resources
Kansas	§ 21-6412	Kansas Department of Wildlife and Parks
Kentucky	§ 525.130	Kentucky Department of Fish and Wildlife Resources
Louisiana	§ 14:102.1	Louisiana Department of Wildlife and Fisheries
Maine	Title 17 § 1031	Maine Department of Inland Fisheries and Wildlife
Maryland	§§ 10-604, 10-606	Maryland Department of Natural Resources
Massachusetts	Ch.272 § 77	Massachusetts Division of Fisheries and Wildlife
Michigan	§§ 750.50, 750.50b	Michigan Department of Natural Resources
Minnesota	§ 343.21	Minnesota Department of Natural Resources
Mississippi	§ 97-41-1	Mississippi Department of Wildlife, Fisheries and Parks
Missouri	§ 578.012	Missouri Department of Conservation
Montana	§ 45-8-211	Montana Fish, Wildlife and Parks
Nebraska	§ 28-1009	Nebraska Game and Parks Commission
Nevada	§ 574.100	Nevada Department of Wildlife
New Hampshire	§ 644:8	New Hampshire Fish and Game Department
New Jersey	§ 4:22-17	New Jersey Fish and Wildlife
New Mexico	§ 30-18-1	New Mexico Department of Game and Fish
New York	§ 353	New York State Dept. of Environmental Conservation
North Carolina	§ 14-360	North Carolina Wildlife Resources Commission
North Dakota	§ 36-21.2-03	North Dakota Game and Fish Department
Ohio	§ 959.13	Ohio Division of Wildlife
Oklahoma	§ 21-1685	Oklahoma Department of Wildlife Conservation
Oregon	§§ 167.315, 167.320, 167.322	Oregon Department of Fish and Wildlife
Pennsylvania	§§ 5533, 5534	Pennsylvania Game Commission
Rhode Island	§ 4-1-2	Rhode Island Department of Environmental Management
South Carolina	§§ 47-1-50, 47-1-70, 47-1-125	South Carolina Department of Natural Resources
South Dakota	§ 40-1-2.4	South Dakota Game, Fish and Parks
Tennessee	§ 39-14-202	Tennessee Wildlife Resources Agency
Texas	§§ 42.09, 42.092	Texas Parks and Wildlife Department
Utah	§ 76-9-301	Utah Division of Wildlife Resources
Vermont	§ 352	Vermont Fish and Wildlife Department
Virginia	§ 3.2-6570	Virginia Department of Wildlife Resources
Washington	§ 16.52.205	Washington Department of Fish and Wildlife
West Virginia	§ 61-8-19	West Virginia Division of Natural Resources
Wisconsin	§§ 951.02, 951.05–951.06, 951.13–951.15	Wisconsin Department of Natural Resources
Wyoming	§§ 6-3-1002, 6-3-1005	Wyoming Game and Fish Department
	Note. The anti-cruelty column identifies the main statutory provisions examined for each jurisdiction. The invasive-species column identifies a principal state agency for invasive-species classifications. The table identifies the principal statutes and agencies rather than every regulation and agency examined.	
