# Supplementary material for: Neither Invasive nor Unprotected: The Legal Status of Muscovy Ducks in the United States
Source: Animals (Basel). 2026 Nov 3;16(21):3472. doi: 10.3390/ani16213472 (PMC13762168; doi:10.3390/ani16213472)
Supplement: Supplementary file 1 [file animals-16-03472-s001.zip › animals-4606646-Supplementary File S1.pdf]

animals

MDPI

Supplementary File S1. State Anti-Cruelty Statutes for Muscovy Ducks in the United States

"Covered" indicates coverage under the listed provisions, subject to statutory exemptions.

ALABAMA
Anti-Cruelty Statute:
Ala. Code §§ 13A-11-14, 13A-11-14.1
Scope of Protection:
any animal
Key Prohibited Conduct:
cruel mistreatment, cruel neglect, kills or injures without good cause any animal belonging to another, infliction of torture, inhumane treatment, gross physical abuse
Muscovy Duck Coverage:
Covered

ALASKA
Anti-Cruelty Statute:
AS §11.61.140
Scope of Protection:
an animal
Key Prohibited Conduct:
inflicts severe or prolonged physical pain or suffering, neglects an animal causing death, neglects causing severe pain or suffering, poisons, kills or injures an animal to intimidate, threaten, or terrorize another person
Muscovy Duck Coverage:
Covered

ARIZONA
Anti-Cruelty Statute:
A.R.S. §13-2910
Scope of Protection:
any animal
Key Prohibited Conduct:
cruel neglect, abandonment, fails to provide medical attention, inflicts unnecessary physical injury, cruel mistreatment, without either legal privilege or consent of the owner, leaves confined causing injury or death
Muscovy Duck Coverage:
Covered

ARKANSAS
Anti-Cruelty Statute:
Ark. Code §5-62-103
Scope of Protection:
any animal
Key Prohibited Conduct:
cruel mistreatment, kills or injures without legal privilege or consent of the owner, abandons an animal, fails to supply food, water, or shelter, carries in a cruel or inhumane manner
Muscovy Duck Coverage:
Covered

CALIFORNIA
Anti-Cruelty Statute:
Cal. Penal Code §597
Scope of Protection:
an animal
Key Prohibited Conduct:
maims, mutilates, tortures, wounds, kills maliciously, deprives of sustenance, cruelly beats, torments
Muscovy Duck Coverage:
Covered

COLORADO
Anti-Cruelty Statute:
CRS §18-9-202
Scope of Protection:
animals
Key Prohibited Conduct:
torments, deprives of necessary sustenance, beats, mistreats, neglects, fails to provide food, needlessly kills
Muscovy Duck Coverage:
Covered

CONNECTICUT
Anti-Cruelty Statute:
§53-247
Scope of Protection:
any animal
Key Prohibited Conduct:
tortures, deprives of necessary sustenance, mutilates, beats, kills unjustifiably
Muscovy Duck Coverage:
Covered

DELAWARE
Anti-Cruelty Statute:
§1325
Scope of Protection:
any animal
Key Prohibited Conduct:
cruel mistreatment, cruel neglect, cruelly or unnecessarily kills or injures any animal, confines an animal unattended, beating, tormenting, abandonment, failure to feed properly, give proper shelter
Muscovy Duck Coverage:
Covered

FLORIDA
Anti-Cruelty Statute:
§828.12
Scope of Protection:
any animal
Key Prohibited Conduct:
torments, deprives of necessary sustenance, deprives of shelter, mutilates, cruel death, carries in a cruel manner, excessive or repeated infliction of unnecessary pain or suffering, unnecessarily kills
Muscovy Duck Coverage:
Covered

GEORGIA
Anti-Cruelty Statute:
§16-12-4
Scope of Protection:
any animal
Key Prohibited Conduct:
causes physical pain, suffering, death, fails to provide food, water, sanitary conditions, ventilation, disfiguring, tortures, maliciously causes death or physical harm
Muscovy Duck Coverage:
Covered

HAWAII
Anti-Cruelty Statute:
§711-1109
Scope of Protection:
animals
Key Prohibited Conduct:
tortures, torments, beats, mutilates, poisons, kills without need, causes substantial bodily injury
Muscovy Duck Coverage:
Covered

IDAHO
Anti-Cruelty Statute:
§25-3504
Scope of Protection:
any animal
Key Prohibited Conduct:
cruel, causes to be cruelly treated, subjects to cruelty, maliciously kill
Muscovy Duck Coverage:
Covered

ILLINOIS
Anti-Cruelty Statute:
510 ILCS 70/3.01, 3.03
Scope of Protection:
animal
Key Prohibited Conduct:
beat, cruelly treat, torment, starve, abuse, abandon, expose, torture, infliction of extreme physical pain
Muscovy Duck Coverage:
Covered

INDIANA
Anti-Cruelty Statute:
IC § 35-46-3-12
Scope of Protection:
any animal
Key Prohibited Conduct:
abuses, cruelty, tortures, mutilates, kills without consent of owner
Muscovy Duck Coverage:
Covered

IOWA
Anti-Cruelty Statute:
§§717.1A, 717.2, 717B.1–717B.3A
Scope of Protection:
animals
Key Prohibited Conduct:
injures, maims, disfigures, destroys, poisoning, fails to supply food, fails to supply water, fails to provide shelter
Muscovy Duck Coverage:
Covered

KANSAS
Anti-Cruelty Statute:
§21-6412
Scope of Protection:
any animal
Key Prohibited Conduct:
maliciously killing, injuring, maiming, torturing, burning, mutilating, abandoning, failing to provide food, water, or protection
Muscovy Duck Coverage:
Covered

KENTUCKY
Anti-Cruelty Statute:
§525.130
Scope of Protection:
any animal
Key Prohibited Conduct:
subjects to mistreatment, abandonment, causing to fight, mutilation, beating, torturing, tormenting, poisons, failing to provide food, drink, or space
Muscovy Duck Coverage:
Covered

LOUISIANA
Anti-Cruelty Statute:
§14:102.1
Scope of Protection:
living animal
Key Prohibited Conduct:
torments, beats, injures, abandons, impounds, confines, tortures, maims, mutilates, fails to provide food, drink, or shelter, causes unnecessary or unjustifiable physical pain, suffering, or death, maliciously killed
Muscovy Duck Coverage:
Covered

MAINE
Anti-Cruelty Statute:
Title 17 §1031
Scope of Protection:
an animal
Key Prohibited Conduct:
kills or attempts to kill without the consent of the owner or legal privilege, injures, tortures, torments, abandons, beats, mutilates, poisons, deprives of sustenance
Muscovy Duck Coverage:
Covered

MARYLAND
Anti-Cruelty Statute:
§§ 10-604, 10-606
Scope of Protection:
an animal
Key Prohibited Conduct:
deprive of sustenance, inflict suffering, inflict pain, fail to provide food, care, drink, or air, intentionally mutilates, tortures, cruelly beats, cruelly kills
Muscovy Duck Coverage:
Covered

MASSACHUSETTS
Anti-Cruelty Statute:
Ch.272 §77
Scope of Protection:
any animal
Key Prohibited Conduct:
tortures, torments, deprives of sustenance, cruelly beats, mutilates or kills
Muscovy Duck Coverage:
Covered

MICHIGAN
Anti-Cruelty Statute:
§§750.50, 750.50b
Scope of Protection:
animal
Key Prohibited Conduct:
fail to provide care, beat, abandon, allow neglect, pain, knowingly kill, torture, mutilate, maim, or disfigure
Muscovy Duck Coverage:
Covered

MINNESOTA
Anti-Cruelty Statute:
§343.21
Scope of Protection:
any animal
Key Prohibited Conduct:
torture, beat, neglect, deprive of necessary food, water, or shelter, unjustifiably injure, maim, mutilate, or kill
Muscovy Duck Coverage:
Covered

MISSISSIPPI
Anti-Cruelty Statute:
§97-41-1
Scope of Protection:
any living creature
Key Prohibited Conduct:
torture, torment, unjustifiably injure, deprive of necessary sustenance, food or drink, cruelly beat, needlessly mutilate or kill
Muscovy Duck Coverage:
Covered

MISSOURI
Anti-Cruelty Statute:
§578.012
Scope of Protection:
an animal
Key Prohibited Conduct:
causes injury or suffering, kills an animal in any manner not allowed, fails to provide adequate care, torture, mutilation
Muscovy Duck Coverage:
Covered

MONTANA
Anti-Cruelty Statute:
§45-8-211
Scope of Protection:
an animal
Key Prohibited Conduct:
subjects to mistreatment or neglect, beating, tormenting, torturing, injuring, killing, confining in a cruel manner, failing to provide food or water
Muscovy Duck Coverage:
Covered

NEBRASKA
Anti-Cruelty Statute:
§28-1009
Scope of Protection:
any animal
Key Prohibited Conduct:
abandons, neglects, mistreats, torture, beating, mutilation
Muscovy Duck Coverage:
Covered

NEVADA
Anti-Cruelty Statute:
§574.100
Scope of Protection:
an animal
Key Prohibited Conduct:
torture, beat, injure, unjustifiably maim, mutilate, or kill, neglect, deprive of food, drink, or sustenance
Muscovy Duck Coverage:
Covered

NEW HAMPSHIRE
Anti-Cruelty Statute:
§644:8
Scope of Protection:
animal
Key Prohibited Conduct:
deprives of care, deprives of sustenance, deprives of shelter, beats, whips, tortures, mutilates, mistreats, abuses, misuses, transports in injurious manner
Muscovy Duck Coverage:
Covered

NEW JERSEY
Anti-Cruelty Statute:
§4:22-17
Scope of Protection:
a living animal
Key Prohibited Conduct:
abuse, needlessly kill, inflict cruelty, leave unattended, fail to provide care, torment, torture, maim, hang, poison, beat
Muscovy Duck Coverage:
Covered

NEW MEXICO
Anti-Cruelty Statute:
§30-18-1
Scope of Protection:
animals
Key Prohibited Conduct:
mistreating, injuring, killing without lawful justification, tormenting, abandoning, failing to provide necessary sustenance, torturing, mutilating, poisoning
Muscovy Duck Coverage:
Covered

NEW YORK
Anti-Cruelty Statute:
§353
Scope of Protection:
any animal
Key Prohibited Conduct:
tortures, beats, unjustifiably injures, maims, mutilates or kills, deprives of sustenance, food, or drink, neglects
Muscovy Duck Coverage:
Covered

NORTH CAROLINA
Anti-Cruelty Statute:
§14-360
Scope of Protection:
any animal
Key Prohibited Conduct:
wound, injure, torment, maliciously kill, deprive of necessary sustenance, torture, mutilate
Muscovy Duck Coverage:
Covered

NORTH DAKOTA
Anti-Cruelty Statute:
§36-21.2-03
Scope of Protection:
any animal
Key Prohibited Conduct:
breaking bones, causing prolonged impairment, mutilating, physically torturing
Muscovy Duck Coverage:
Covered

OHIO
Anti-Cruelty Statute:
§959.13
Scope of Protection:
an animal
Key Prohibited Conduct:
tortures, cruelly beats, needlessly mutilate or kill, confines without food, water, or shelter
Muscovy Duck Coverage:
Covered

OKLAHOMA
Anti-Cruelty Statute:
§21-1685
Scope of Protection:
any animal
Key Prohibited Conduct:
maliciously torture, destroy, or kill, beat, injure, maim, mutilate, deprive of food, drink, or shelter
Muscovy Duck Coverage:
Covered

OREGON
Anti-Cruelty Statute:
§§167.315, 167.320, 167.322
Scope of Protection:
any animal
Key Prohibited Conduct:
causes serious physical injury or death, tortures
Muscovy Duck Coverage:
Covered

PENNSYLVANIA
Anti-Cruelty Statute:
§§5533, 5534
Scope of Protection:
an animal
Key Prohibited Conduct:
recklessly illtreats, beats, abandons, or abuses an animal, tortures, causes serious bodily injury or death
Muscovy Duck Coverage:
Covered

RHODE ISLAND
Anti-Cruelty Statute:
§4-1-2
Scope of Protection:
any animal
Key Prohibited Conduct:
tortures, torments, deprives of sustenance, cruelly beats, mutilates, or kills, fails to provide food or drink
Muscovy Duck Coverage:
Covered

SOUTH CAROLINA
Anti-Cruelty Statute:
§§47-1-50, 47-1-70, 47-1-125
Scope of Protection:
any animal
Key Prohibited Conduct:
carry in an unnecessarily cruel or inhumane manner, abandons without securing another owner or providing necessities of life, dye or color artificially
Muscovy Duck Coverage:
Covered

SOUTH DAKOTA
Anti-Cruelty Statute:
§40-1-2.4
Scope of Protection:
any animal
Key Prohibited Conduct:
subject to cruelty
Muscovy Duck Coverage:
Covered

TENNESSEE
Anti-Cruelty Statute:
§39-14-202
Scope of Protection:
an animal
Key Prohibited Conduct:
tortures, maims, fails to provide food, fails to provide water, fails to provide care, fails to provide shelter, abandons
Muscovy Duck Coverage:
Covered

TEXAS
Anti-Cruelty Statute:
§§42.09, 42.092
Scope of Protection:
an animal
Key Prohibited Conduct:
tortures or kills in a cruel manner, causes serious bodily injury, administers poison, fails to provide food, water, care, or shelter, abandons, confines in cruel manner
Muscovy Duck Coverage:
Covered

UTAH
Anti-Cruelty Statute:
§76-9-301
Scope of Protection:
an animal
Key Prohibited Conduct:
fails to provide food, care, or shelter, abandons, confines in cruel manner, injures, tortures, administers poison, kills without legal privilege
Muscovy Duck Coverage:
Covered

VERMONT
Anti-Cruelty Statute:
§352
Scope of Protection:
any animal
Key Prohibited Conduct:
kills or attempts to kill without legal authority or consent of owner, tortures, torments, abandons, administers poison, harms, mutilates, exposes poison, deprives of food or water
Muscovy Duck Coverage:
Covered

VIRGINIA
Anti-Cruelty Statute:
§3.2-6570
Scope of Protection:
any animal
Key Prohibited Conduct:
ill-treats, abandons, tortures, willfully inflicts inhumane injury or pain, cruelly or unnecessarily beats, maims, mutilates, kills, deprives of necessary food, drink, or shelter
Muscovy Duck Coverage:
Covered

WASHINGTON
Anti-Cruelty Statute:
§16.52.205
Scope of Protection:
any animal
Key Prohibited Conduct:
inflicts substantial pain, causes injury, kills causing undue suffering, starves, dehydrates, suffocates, exposes to heat, exposes to cold, causes pain, causes death
Muscovy Duck Coverage:
Covered

WEST VIRGINIA
Anti-Cruelty Statute:
§61-8-19
Scope of Protection:
any animal
Key Prohibited Conduct:
mistreat, abandon, withhold sustenance, shelter, or medical treatment, abandon to die, leave unattended, bait, harass, intentionally tortures, mutilates, maliciously kills
Muscovy Duck Coverage:
Covered

WISCONSIN
Anti-Cruelty Statute:
§§951.02, 951.05–951.06, 951.13–951.15
Scope of Protection:
any animal
Key Prohibited Conduct:
treat in cruel manner, kill, poison, shoot, wound, fail to supply food, water, or shelter, abandon
Muscovy Duck Coverage:
Covered

WYOMING
Anti-Cruelty Statute:
§§6-3-1002, 6-3-1005
Scope of Protection:
any animal
Key Prohibited Conduct:
unnecessarily beats or injures, fails to provide food, drink, or protection, abandons
Muscovy Duck Coverage:
Covered
