# Supplementary material for: Neither Invasive nor Unprotected: The Legal Status of Muscovy Ducks in the United States
Source: Animals (Basel). 2026 Nov 3;16(21):3472. doi: 10.3390/ani16213472 (PMC13762168; doi:10.3390/ani16213472)
Supplement: Supplementary file 1 [file animals-16-03472-s001.zip › animals-4606646-Supplementary File S2.pdf]

animals

Supplementary File S2: State Invasive-Species Classification and Assessment Status for Muscovy Ducks in the United States

ALABAMA
State Agency:
Alabama Department of Conservation and Natural Resources
Invasive-Species Classification:
No Muscovy Duck-specific invasive-species listing identified.
Assessment of Harm:
No Muscovy Duck-specific assessment of actual or likely harm identified.
Formal Invasive-Species Status:
Not formally classified as invasive.

ALASKA
State Agency:
Alaska Department of Fish and Game
Invasive-Species Classification:
No Muscovy Duck-specific invasive-species listing identified.
Assessment of Harm:
No Muscovy Duck-specific assessment of actual or likely harm identified.
Formal Invasive-Species Status:
Not formally classified as invasive.

ARIZONA
State Agency:
Arizona Game and Fish Department
Invasive-Species Classification:
No Muscovy Duck-specific invasive-species listing identified.
Assessment of Harm:
No Muscovy Duck-specific assessment of actual or likely harm identified.
Formal Invasive-Species Status:
Not formally classified as invasive.

ARKANSAS
State Agency:
Arkansas Game and Fish Commission
Invasive-Species Classification:
No Muscovy Duck-specific invasive-species listing identified.
Assessment of Harm:
No Muscovy Duck-specific assessment of actual or likely harm identified.
Formal Invasive-Species Status:
Not formally classified as invasive.

CALIFORNIA
State Agency:
California Department of Fish and Wildlife
Invasive-Species Classification:
No Muscovy Duck-specific invasive-species listing identified.
Assessment of Harm:
No Muscovy Duck-specific assessment of actual or likely harm identified.
Formal Invasive-Species Status:
Not formally classified as invasive.

COLORADO
State Agency:
Colorado Parks and Wildlife
Invasive-Species Classification:
No Muscovy Duck-specific invasive-species listing identified.
Assessment of Harm:
No Muscovy Duck-specific assessment of actual or likely harm identified.
Formal Invasive-Species Status:
Not formally classified as invasive.

CONNECTICUT
State Agency:
Connecticut Department of Energy and Environmental Protection
Invasive-Species Classification:
No Muscovy Duck-specific invasive-species listing identified.
Assessment of Harm:
No Muscovy Duck-specific assessment of actual or likely harm identified.
Formal Invasive-Species Status:
Not formally classified as invasive.

DELAWARE
State Agency:
Delaware Division of Fish and Wildlife
Invasive-Species Classification:
No Muscovy Duck-specific invasive-species listing identified.
Assessment of Harm:
No Muscovy Duck-specific assessment of actual or likely harm identified.
Formal Invasive-Species Status:
Not formally classified as invasive.

FLORIDA
State Agency:
Florida Fish and Wildlife Conservation Commission
Invasive-Species Classification:
No Muscovy Duck-specific invasive-species listing identified.
Assessment of Harm:
No Muscovy Duck-specific assessment of actual or likely harm identified.
Formal Invasive-Species Status:
Not formally classified as invasive.

GEORGIA
State Agency:
Georgia Wildlife Resources Division
Invasive-Species Classification:
No Muscovy Duck-specific invasive-species listing identified.
Assessment of Harm:
No Muscovy Duck-specific assessment of actual or likely harm identified.
Formal Invasive-Species Status:
Not formally classified as invasive.

HAWAII
State Agency:
Hawaii Department of Land and Natural Resources
Invasive-Species Classification:
No Muscovy Duck-specific invasive-species listing identified.
Assessment of Harm:
No Muscovy Duck-specific assessment of actual or likely harm identified.
Formal Invasive-Species Status:
Not formally classified as invasive.

IDAHO
State Agency:
Idaho Fish and Game
Invasive-Species Classification:
No Muscovy Duck-specific invasive-species listing identified.
Assessment of Harm:
No Muscovy Duck-specific assessment of actual or likely harm identified.
Formal Invasive-Species Status:
Not formally classified as invasive.

ILLINOIS
State Agency:
Illinois Department of Natural Resources
Invasive-Species Classification:
No Muscovy Duck-specific invasive-species listing identified.
Assessment of Harm:
No Muscovy Duck-specific assessment of actual or likely harm identified.
Formal Invasive-Species Status:
Not formally classified as invasive.

INDIANA
State Agency:
Indiana Division of Fish and Wildlife
Invasive-Species Classification:
No Muscovy Duck-specific invasive-species listing identified.
Assessment of Harm:
No Muscovy Duck-specific assessment of actual or likely harm identified.
Formal Invasive-Species Status:
Not formally classified as invasive.

IOWA
State Agency:
Iowa Department of Natural Resources
Invasive-Species Classification:
No Muscovy Duck-specific invasive-species listing identified.
Assessment of Harm:
No Muscovy Duck-specific assessment of actual or likely harm identified.
Formal Invasive-Species Status:
Not formally classified as invasive.

KANSAS
State Agency:
Kansas Department of Wildlife and Parks
Invasive-Species Classification:
No Muscovy Duck-specific invasive-species listing identified.
Assessment of Harm:
No Muscovy Duck-specific assessment of actual or likely harm identified.
Formal Invasive-Species Status:
Not formally classified as invasive.

KENTUCKY
State Agency:
Kentucky Department of Fish and Wildlife Resources
Invasive-Species Classification:
No Muscovy Duck-specific invasive-species listing identified.
Assessment of Harm:
No Muscovy Duck-specific assessment of actual or likely harm identified.
Formal Invasive-Species Status:
Not formally classified as invasive.

LOUISIANA
State Agency:
Louisiana Department of Wildlife and Fisheries
Invasive-Species Classification:
Feral Muscovy Duck is included in Tier 3 of the 2025 Louisiana Wildlife Action Plan invasive-species list, which includes species with limited or poorly known impacts.
Assessment of Harm:
LDWF confirmed that no formal Muscovy Duck-specific assessment of harm has been conducted.
Formal Invasive-Species Status:
Formal listing as invasive, lacking formal science-based substantiation of harm in the state.

MAINE
State Agency:
Maine Department of Inland Fisheries and Wildlife
Invasive-Species Classification:
No Muscovy Duck-specific invasive-species listing identified.
Assessment of Harm:
No Muscovy Duck-specific assessment of actual or likely harm identified.
Formal Invasive-Species Status:
Not formally classified as invasive.

MARYLAND
State Agency:
Maryland Department of Natural Resources
Invasive-Species Classification:
No Muscovy Duck-specific invasive-species listing identified.
Assessment of Harm:
No Muscovy Duck-specific assessment of actual or likely harm identified.
Formal Invasive-Species Status:
Not formally classified as invasive.

MASSACHUSETTS
State Agency:
Massachusetts Division of Fisheries and Wildlife
Invasive-Species Classification:
No Muscovy Duck-specific invasive-species listing identified.
Assessment of Harm:
No Muscovy Duck-specific assessment of actual or likely harm identified.
Formal Invasive-Species Status:
Not formally classified as invasive.

MICHIGAN
State Agency:
Michigan Department of Natural Resources
Invasive-Species Classification:
No Muscovy Duck-specific invasive-species listing identified.
Assessment of Harm:
No Muscovy Duck-specific assessment of actual or likely harm identified.
Formal Invasive-Species Status:
Not formally classified as invasive.

MINNESOTA
State Agency:
Minnesota Department of Natural Resources
Invasive-Species Classification:
No Muscovy Duck-specific invasive-species listing identified.
Assessment of Harm:
No Muscovy Duck-specific assessment of actual or likely harm identified.
Formal Invasive-Species Status:
Not formally classified as invasive.

MISSISSIPPI
State Agency:
Mississippi Department of Wildlife, Fisheries and Parks
Invasive-Species Classification:
No Muscovy Duck-specific invasive-species listing identified.
Assessment of Harm:
No Muscovy Duck-specific assessment of actual or likely harm identified.
Formal Invasive-Species Status:
Not formally classified as invasive.

MISSOURI
State Agency:
Missouri Department of Conservation
Invasive-Species Classification:
No Muscovy Duck-specific invasive-species listing identified.
Assessment of Harm:
No Muscovy Duck-specific assessment of actual or likely harm identified.
Formal Invasive-Species Status:
Not formally classified as invasive.

MONTANA
State Agency:
Montana Fish, Wildlife and Parks
Invasive-Species Classification:
No Muscovy Duck-specific invasive-species listing identified.
Assessment of Harm:
No Muscovy Duck-specific assessment of actual or likely harm identified.
Formal Invasive-Species Status:
Not formally classified as invasive.

NEBRASKA
State Agency:
Nebraska Game and Parks Commission
Invasive-Species Classification:
No Muscovy Duck-specific invasive-species listing identified.
Assessment of Harm:
No Muscovy Duck-specific assessment of actual or likely harm identified.
Formal Invasive-Species Status:
Not formally classified as invasive.

NEVADA
State Agency:
Nevada Department of Wildlife
Invasive-Species Classification:
No Muscovy Duck-specific invasive-species listing identified.
Assessment of Harm:
No Muscovy Duck-specific assessment of actual or likely harm identified.
Formal Invasive-Species Status:
Not formally classified as invasive.

NEW HAMPSHIRE
State Agency:
New Hampshire Fish and Game Department
Invasive-Species Classification:
No Muscovy Duck-specific invasive-species listing identified.
Assessment of Harm:
No Muscovy Duck-specific assessment of actual or likely harm identified.
Formal Invasive-Species Status:
Not formally classified as invasive.

NEW JERSEY
State Agency:
New Jersey Fish and Wildlife
Invasive-Species Classification:
No Muscovy Duck-specific invasive-species listing identified.
Assessment of Harm:
No Muscovy Duck-specific assessment of actual or likely harm identified.
Formal Invasive-Species Status:
Not formally classified as invasive.

NEW MEXICO
State Agency:
New Mexico Department of Game and Fish
Invasive-Species Classification:
No Muscovy Duck-specific invasive-species listing identified.
Assessment of Harm:
No Muscovy Duck-specific assessment of actual or likely harm identified.
Formal Invasive-Species Status:
Not formally classified as invasive.

NEW YORK
State Agency:
New York State Department of Environmental Conservation
Invasive-Species Classification:
Muscovy Duck is listed as a regulated invasive species under 6 NYCRR § 575.4.
Assessment of Harm:
The ecological and socio-economic assessments lacked scientific evidence establishing the requisite harm in the state.
Formal Invasive-Species Status:
Formal listing as invasive, lacking formal science-based substantiation of harm in the state.

NORTH CAROLINA
State Agency:
North Carolina Wildlife Resources Commission
Invasive-Species Classification:
No Muscovy Duck-specific invasive-species listing identified.
Assessment of Harm:
No Muscovy Duck-specific assessment of actual or likely harm identified.
Formal Invasive-Species Status:
Not formally classified as invasive.

NORTH DAKOTA
State Agency:
North Dakota Game and Fish Department
Invasive-Species Classification:
No Muscovy Duck-specific invasive-species listing identified.
Assessment of Harm:
No Muscovy Duck-specific assessment of actual or likely harm identified.
Formal Invasive-Species Status:
Not formally classified as invasive.

OHIO
State Agency:
Ohio Division of Wildlife
Invasive-Species Classification:
No Muscovy Duck-specific invasive-species listing identified.
Assessment of Harm:
No Muscovy Duck-specific assessment of actual or likely harm identified.
Formal Invasive-Species Status:
Not formally classified as invasive.

OKLAHOMA
State Agency:
Oklahoma Department of Wildlife Conservation
Invasive-Species Classification:
No Muscovy Duck-specific invasive-species listing identified.
Assessment of Harm:
No Muscovy Duck-specific assessment of actual or likely harm identified.
Formal Invasive-Species Status:
Not formally classified as invasive.

OREGON
State Agency:
Oregon Department of Fish and Wildlife
Invasive-Species Classification:
No Muscovy Duck-specific invasive-species listing identified.
Assessment of Harm:
No Muscovy Duck-specific assessment of actual or likely harm identified.
Formal Invasive-Species Status:
Not formally classified as invasive.

PENNSYLVANIA
State Agency:
Pennsylvania Game Commission
Invasive-Species Classification:
No Muscovy Duck-specific invasive-species listing identified.
Assessment of Harm:
No Muscovy Duck-specific assessment of actual or likely harm identified.
Formal Invasive-Species Status:
Not formally classified as invasive.

RHODE ISLAND
State Agency:
Rhode Island Department of Environmental Management
Invasive-Species Classification:
No Muscovy Duck-specific invasive-species listing identified.
Assessment of Harm:
No Muscovy Duck-specific assessment of actual or likely harm identified.
Formal Invasive-Species Status:
Not formally classified as invasive.

SOUTH CAROLINA
State Agency:
South Carolina Department of Natural Resources
Invasive-Species Classification:
No Muscovy Duck-specific invasive-species listing identified.
Assessment of Harm:
No Muscovy Duck-specific assessment of actual or likely harm identified.
Formal Invasive-Species Status:
Not formally classified as invasive.

SOUTH DAKOTA
State Agency:
South Dakota Game, Fish and Parks
Invasive-Species Classification:
No Muscovy Duck-specific invasive-species listing identified.
Assessment of Harm:
No Muscovy Duck-specific assessment of actual or likely harm identified.
Formal Invasive-Species Status:
Not formally classified as invasive.

TENNESSEE
State Agency:
Tennessee Wildlife Resources Agency
Invasive-Species Classification:
No Muscovy Duck-specific invasive-species listing identified.
Assessment of Harm:
No Muscovy Duck-specific assessment of actual or likely harm identified.
Formal Invasive-Species Status:
Not formally classified as invasive.

TEXAS
State Agency:
Texas Parks and Wildlife Department
Invasive-Species Classification:
No Muscovy Duck-specific invasive-species listing identified.
Assessment of Harm:
No Muscovy Duck-specific assessment of actual or likely harm identified.
Formal Invasive-Species Status:
Not formally classified as invasive.

UTAH
State Agency:
Utah Division of Wildlife Resources
Invasive-Species Classification:
No Muscovy Duck-specific invasive-species listing identified.
Assessment of Harm:
No Muscovy Duck-specific assessment of actual or likely harm identified.
Formal Invasive-Species Status:
Not formally classified as invasive.

VERMONT
State Agency:
Vermont Fish and Wildlife Department
Invasive-Species Classification:
No Muscovy Duck-specific invasive-species listing identified.
Assessment of Harm:
No Muscovy Duck-specific assessment of actual or likely harm identified.
Formal Invasive-Species Status:
Not formally classified as invasive.

VIRGINIA
State Agency:
Virginia Department of Wildlife Resources
Invasive-Species Classification:
No Muscovy Duck-specific invasive-species listing identified.
Assessment of Harm:
No Muscovy Duck-specific assessment of actual or likely harm identified.
Formal Invasive-Species Status:
Not formally classified as invasive.

WASHINGTON
State Agency:
Washington Department of Fish and Wildlife
Invasive-Species Classification:
No Muscovy Duck-specific invasive-species listing identified.
Assessment of Harm:
No Muscovy Duck-specific assessment of actual or likely harm identified.
Formal Invasive-Species Status:
Not formally classified as invasive.

WEST VIRGINIA
State Agency:
West Virginia Division of Natural Resources
Invasive-Species Classification:
No Muscovy Duck-specific invasive-species listing identified.
Assessment of Harm:
No Muscovy Duck-specific assessment of actual or likely harm identified.
Formal Invasive-Species Status:
Not formally classified as invasive.

WISCONSIN
State Agency:
Wisconsin Department of Natural Resources
Invasive-Species Classification:
No Muscovy Duck-specific invasive-species listing identified.
Assessment of Harm:
No Muscovy Duck-specific assessment of actual or likely harm identified.
Formal Invasive-Species Status:
Not formally classified as invasive.

WYOMING
State Agency:
Wyoming Game and Fish Department
Invasive-Species Classification:
No Muscovy Duck-specific invasive-species listing identified.
Assessment of Harm:
No Muscovy Duck-specific assessment of actual or likely harm identified.
Formal Invasive-Species Status:
Not formally classified as invasive.
